# Prediction of humeral torsion from proximal humerus computed tomography is enabled by three-dimensional estimation of the transepicondylar axis using deep learning

**DOI:** 10.1016/j.jseint.2026.101775

**Published:** 2026-08-05

**Authors:** Alexandra Oswald, Hanspeter Hess, Michael Schär, Matthias A. Zumstein, Kate Gerber

**Affiliations:** a Department of Orthopaedic Surgery and Traumatology, Inselspital, Bern University Hospital, University of Bern, Bern, Switzerland; b Shoulder, Elbow and Orthopaedic Sports Medicine, Orthopaedics Sonnenhof, Bern, Switzerland; c Shoulder, Elbow Unit, Sportsclinicnumber1, Bern, Switzerland; d Faculty of Medicine, University of Bern, Bern, Switzerland; e Faculty of Medicine, Health and Human Sciences, Macquarie University, Sydney, Australia

**Keywords:** Humeral torsion, Reverse total shoulder arthroplasty, Osteoarthritis, Proximal humeral morphology, Deep learning prediction, Transepicondylar axis, Surgical planning

## Abstract

**Background:**

Humeral torsion is an important factor for reverse total shoulder arthroplasty planning, yet its measurement requires elbow imaging frequently absent from clinical computed tomography (CT) scans. We hypothesized that a deep learning approach could accurately predict humeral torsion from proximal humerus morphology alone.

**Methods:**

CT-based segmentations from 581 shoulders were used to train and evaluate a deep learning model within the nnU-Net framework. The transepicondylar axis was represented as a full 3-dimensional plane extending throughout the imaging volume. The model was trained on 478 cases and tested on 103, with ground-truth torsion derived from elbow segmentations. Robustness to osteophytes and reduced fields of view was assessed.

**Results:**

The model achieved a mean absolute torsion prediction error of 5.35° ± 3.96° and a strong linear correlation with ground-truth measurements (R = 0.85), outperforming prior proximal landmark-based approaches (R = 0.33–0.42). Performance was robust to osteophytes (mean error: 6.19° ± 3.95°) and was maintained with proximal field of view reductions down to 25% of humeral length, beyond which accuracy progressively declined.

**Conclusion:**

Deep learning can predict humeral torsion directly from proximal humerus morphology with clinically relevant accuracy, reducing the need for distal elbow imaging. These findings support the hypothesis that torsional alignment is largely encoded in the proximal epiphysis and offers a practical solution for reverse total shoulder arthroplasty planning when full-length CT is unavailable.

Humeral torsion, defined as the axial rotation of the humerus, has a significant impact on the position of the glenohumeral joint center,^27^ the potential development of osteoarthritis,^22^ the stability of the joint,^6^ the transverse force couple muscle volumes,^1^ and the range of internal and external humeral rotation.^16^^,^^23^ In general, an increased humeral antetorsion allows for more impingement-free internal rotation, whereas retrotorsion improves external rotation capacity.^27^ These biomechanical effects are particularly important in reverse total shoulder arthroplasty (rTSA), in which the native concave glenoid is replaced with a convex implant, and the humerus receives the concave socket, restoring shoulder stability even in the presence of a rotator cuff pathology by improving the deltoid moment arm and reducing the cumulative deltoid force.^10^ Although reported mean healthy humeral torsion values vary widely (range, 12° to 36°^8^^,^^11^^,^^22^), several studies have suggested that maintaining native torsion in rTSA provides the best balance between internal and external rotation.^3^^,^^20^ Additionally, native humeral torsion can improve congruency between the implant and native epiphysis, thereby reducing the risk of fractures of the greater and lesser tuberosities.^3^

Humeral torsion is calculated from computed tomography (CT) scans, as the angle between the humeral axis and the elbow's transepicondylar axis projected on the transverse plane of the humeral head.^4^ However, many shoulder CT scans do not include the elbow within the field of view (FOV), which limits the measurement of humeral torsion in daily clinical routine. Mechanobiological evidence suggests that torsional alignment of the humerus develops primarily at the proximal epiphysis under the influence of asymmetric muscular loading during growth.^17^^,^^25^ It is therefore plausible that the proximal humerus encodes the torsional characteristics of the entire bone.

Prior attempts at inferring torsion from proximal anatomy have not achieved sufficient reliability.^8^^,^^14^ However, this is likely due to reliance on use of the bicipital groove, which Balg et al^2^ showed varies along the humeral axis, resulting in inaccurate humeral torsion measurements.

Recent advances in deep learning have enabled accurate characterization of glenoid morphology and prediction of scapular axes using segmentations derived from magnetic resonance imaging with an incomplete FOV of the scapula.^12^ Given the ability of deep learning models to exploit full three-dimensional (3D) morphology for axes prediction from images despite a reduced FOV, we hypothesize that a geometry-based deep learning approach to humeral transepicondylar axis prediction and, thereby, humeral torsion calculation, based solely on the morphology of the proximal humerus would provide sufficient accuracy for rTSA planning without the need for an additional image of the elbow.

## Methods

**Figure 1:**
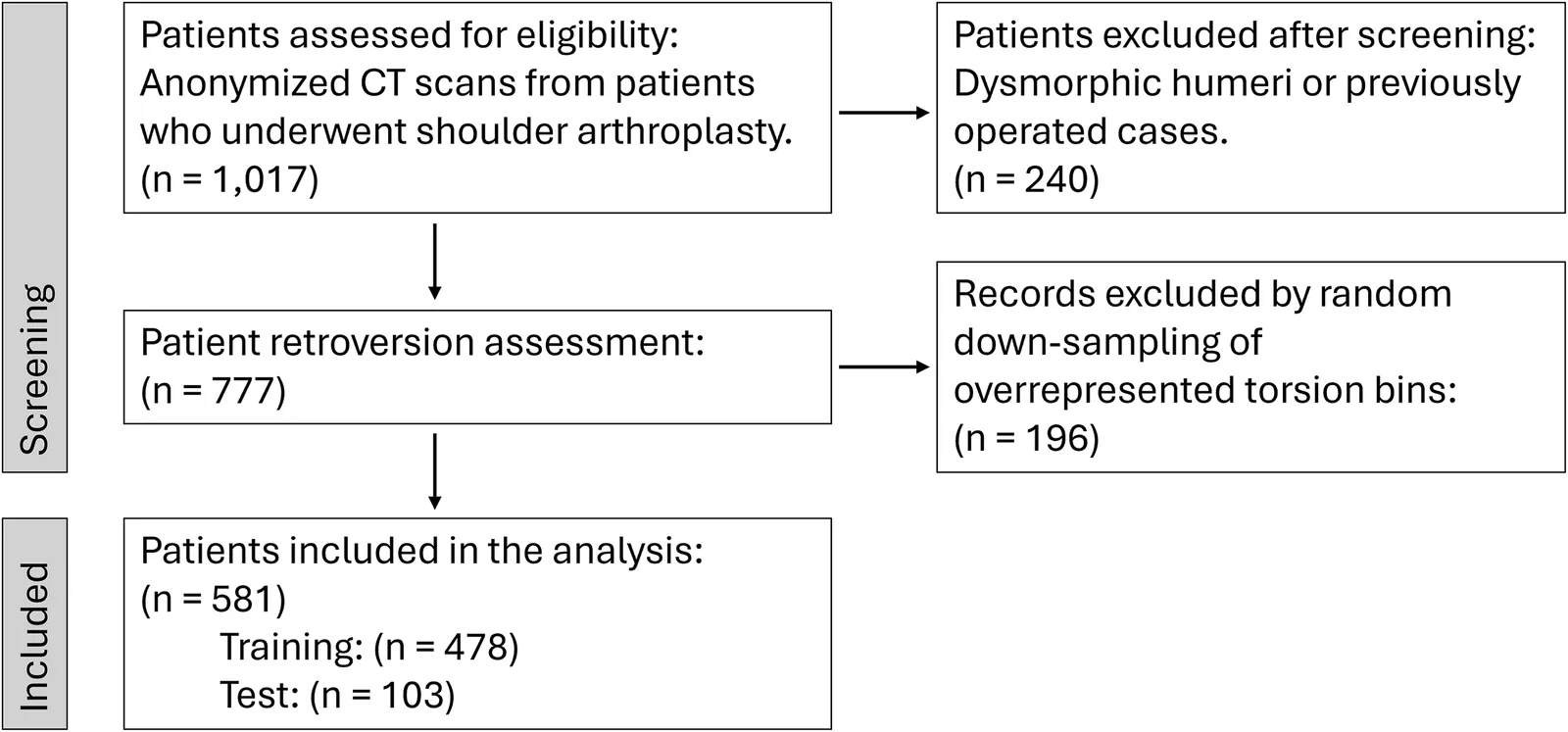
Selection of included patients. *CT*, computed tomography.

This study used anonymized CT data (n = 1,017, in-plane resolution range 0.19 mm-1.0 mm, slice thickness range 0.5mm-2.0 mm) acquired across multiple centers on scanners from 5 vendors (Canon, GE, Philips, Siemens, Toshiba) from patients who had previously undergone shoulder arthroplasty. Cases with extreme dysplastic humeral morphology (n = 120) and prior surgical intervention including implants, cut-planes, or hardware artifacts (n = 92) were excluded following visual inspection of the CT images, resulting in a final cohort of 805 subjects. Patients were stratified into 10° bins based on humeral torsion values spanning −10° to 60° (Table I). To address class imbalance, bins containing more than 125 subjects were randomly downsampled to 125 cases. For bins with fewer than 125 subjects, all cases were retained. Following this procedure, 581 patients were included in the final analysis (Fig. 1).

**Table I:** Patient humeral torsion stratified into 10° bins.

Humeral torsion [°]	Total patients	Down-sampled
(−10, 0)	22	22
(0, 10)	45	45
(10, 20)	108	108
(20, 30)	136	125
(30, 40)	301	125
(40, 50)	134	125
(50, 60)	31	31

Bins containing more than 125 patients were randomly down-sampled to homogenize the distribution.

**Figure 2:**
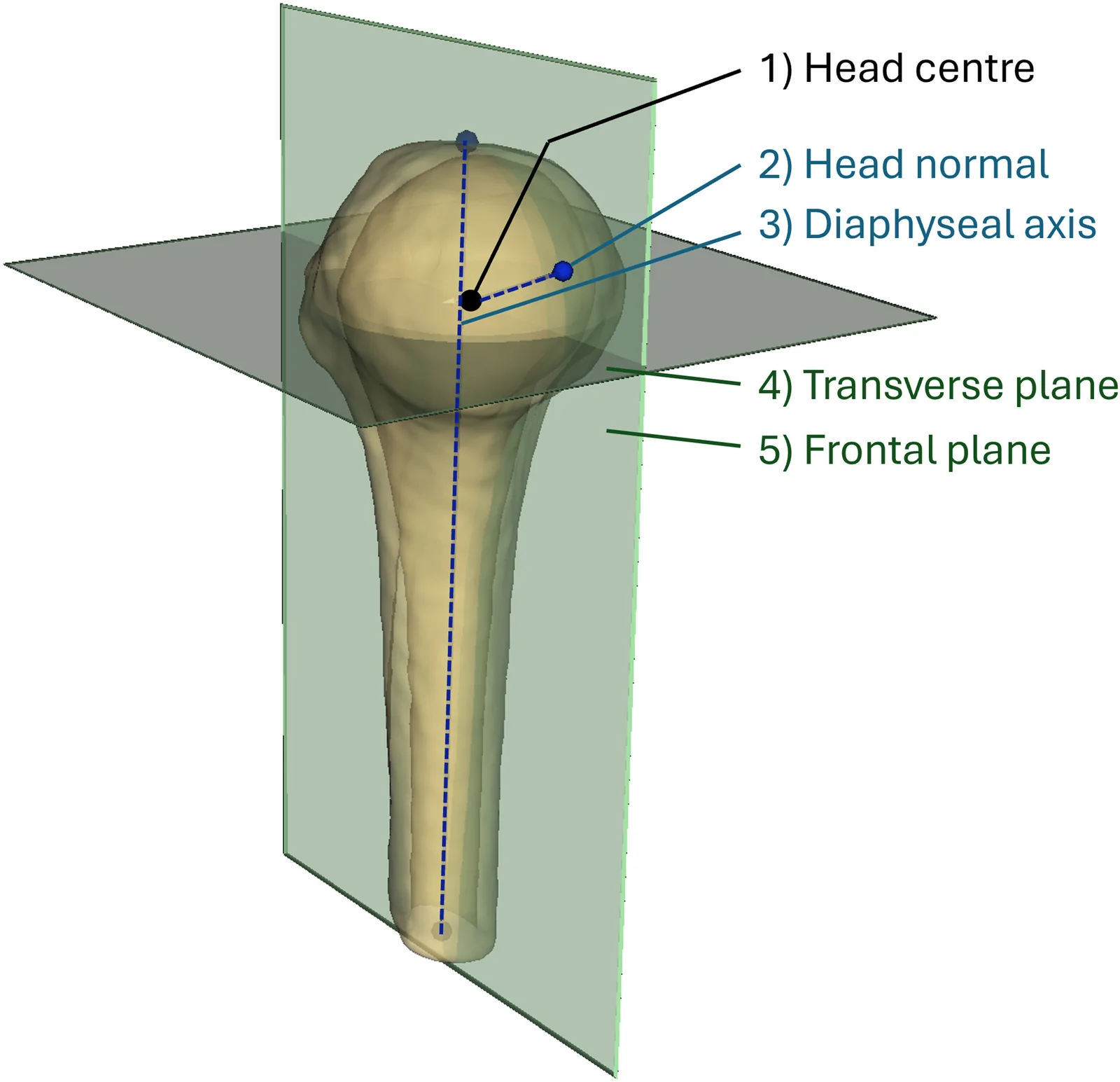
Proximal humeral landmarks. (1) Humeral head center, defined as the center of a best-fit sphere to the humeral head; (2) Humeral head normal, defined as the best-fit orientation of the humeral articular surface; (3) Diaphyseal axis, defined as the best-fit axis through the intramedullary canal; (4) Humeral transverse plane, perpendicular to the diaphyseal axis and passing through the humeral head center; (5) Humeral frontal plane, spanning the diaphyseal axis and humeral head normal.

For each patient, the final dataset comprised of CT-based segmentations of the humerus with and without osteophytes, the elbow segmentation, the scapula segmentation, and landmarks, planes, and axes defined pre-operatively for arthroplasty planning. Relevant landmarks were extracted including the medial and lateral epicondyles of the elbow and the humeral head center (defined as the center of a best-fit sphere to the humeral head). The transepicondylar axis was defined as the axis linking the medal and lateral epicondyles of the elbow. The humeral diaphyseal axis was defined as the best-fit axis through the intramedullary canal, and the humeral head normal as the normal to the plane best representing humeral articular surface orientation, defined by osteophytes at its base. Two planes describing proximal humeral morphology were also defined: the humeral transverse plane, perpendicular to the diaphyseal axis and passing through the humeral head center, and the humeral frontal plane, spanning the diaphyseal axis and humeral head normal (Fig. 2). The glenoid retroversion of the corresponding scapulae within the cohort was calculated according to the Friedman method.^24^

The sphericity of the humeral head was defined as the mean absolute distance of the humeral head surface from the ideal sphere, expressed as a percentage of the sphere radius.^9^ Ground truth humeral torsion for each patient was calculated as the angle between the humeral head normal and the transepicondylar axis projected onto the humeral transverse plane according to Boileau et al.^4^

For automatic transepicondylar axis prediction in the absence of an elbow CT, a deep learning–based approach was implemented using the nnU-Net framework.^13^ The dataset was split into training (N = 478) and test (N = 103) subsets using an 80:20 ratio and ensuring a homogeneous distribution of humeral torsion between the test and training data sets. To reduce underrepresentation during model training, a minimum of 4 patients per 10° torsion bin was enforced in each fold.

**Figure 3:**
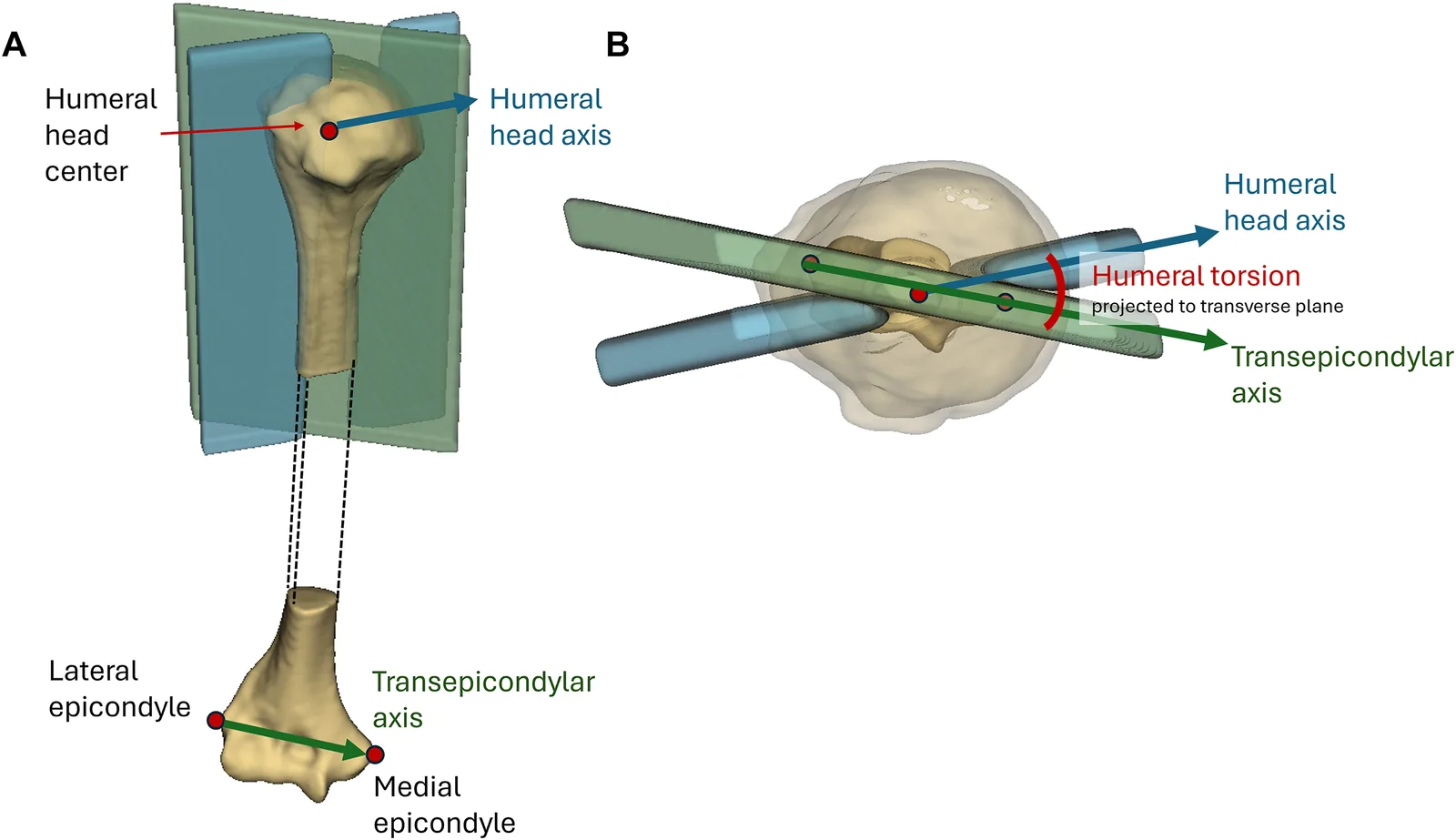
(**A**) Paired training data consisted of the proximal humeral segment and plane segmentations. Green: The epicondylar plane, defined between the humeral head center, the lateral and medial epicondyles. Blue: The humeral normal plane defined parallel to the humeral head normal through the humeral head center. (**B**) Transverse view of the network training planes. The humeral torsion is defined as the angle between the transepicondylar axis and the humeral head normal projected onto the transverse plane.

For preprocessing, all segmentations were aligned to a coordinate system defined by the humeral diaphyseal axis and the humeral head normal projected onto the transverse plane, with the third axis perpendicular to these. Segmentations were then resampled to an isotropic 0.5 mm resolution and resized to 150 × 150 × 300 voxels. To represent the morphology of the transepicondylar axis beyond the FOV of the proximal humerus, a binary mask was generated to define the plane passing through the humeral center point and the epicondylar landmarks. This plane extended through the entire volume with a width of 4 mm. An additional 4 mm binary mask plane, the humeral frontal plane, defined perpendicular to the humeral head normal, was included to provide additional context for the network (Fig. 3). For each plane, the in-plane vectors perpendicular to the normal plane were projected onto the humeral transverse plane to represent the orientation of the transepicondylar axis and the humeral normal axis, respectively.

The paired plane labels and humeral segmentation were used as inputs to train the network to predict both the transepicondylar axis and the humeral head normal, using 5-fold cross-validation over 300 epochs.

Plane prediction accuracy was quantified using the mean absolute angular error between the predicted and ground-truth humeral head normal axes. Because the humeral head normal can be measured directly from the proximal shoulder CT image, the original ground-truth humeral normal axis was used to quantify the humeral torsion. Predicted humeral torsion was defined as the angle between the predicted transepicondylar axis and the ground-truth humeral normal axis. Torsion error was calculated as the mean absolute angular error relative to the ground-truth torsion derived from elbow segmentation, and agreement was assessed using linear regression.

The effect of osteophytes on network performance was evaluated using the same training splits and methods, but with segmentations that included osteophytes.

**Figure 4:**
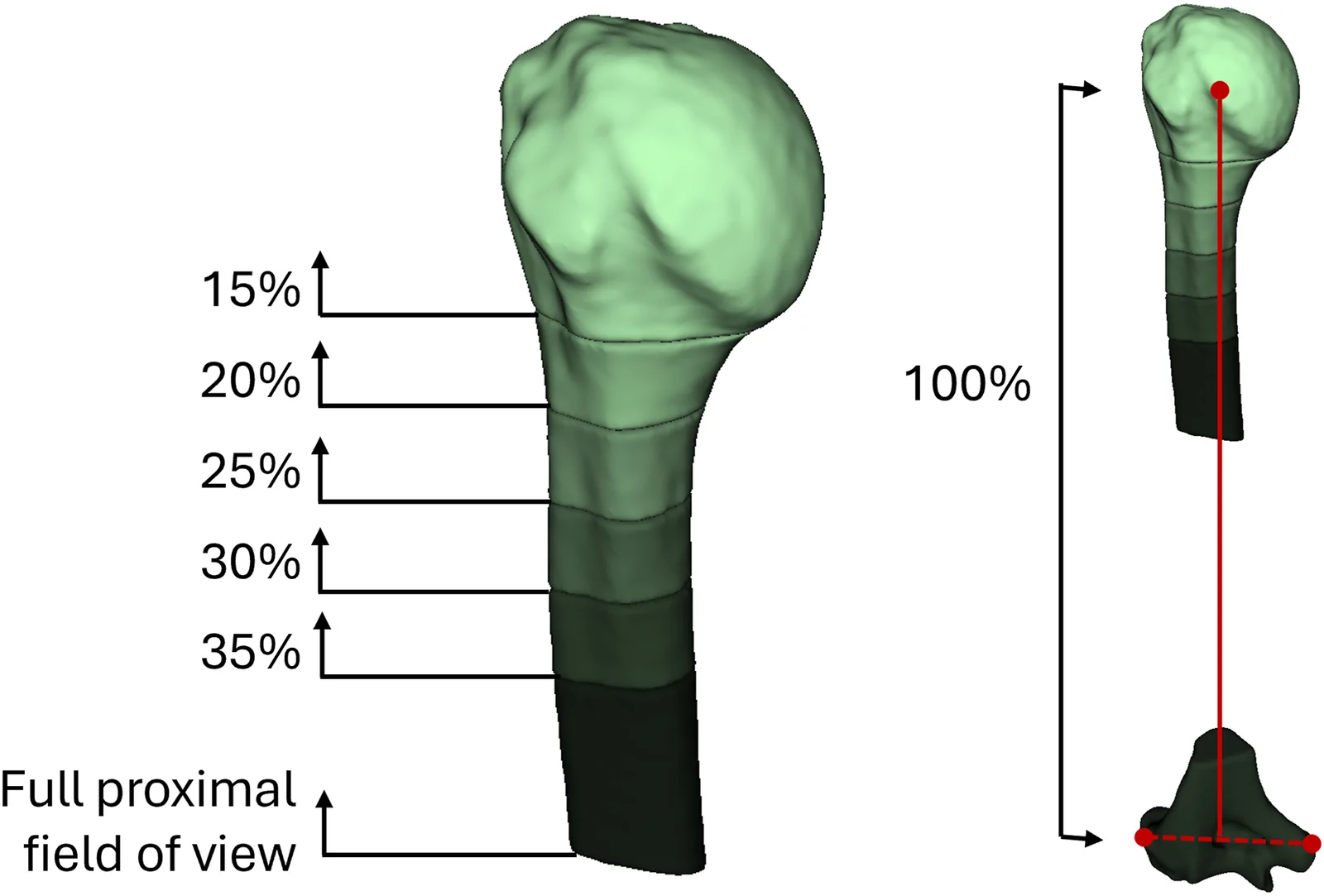
Humeral crops expressed as percentage of full humeral length as defined as the distance between the humeral head center to the mid-point between the medial and lateral epicondyles. Five crops were defined at 15, 20, 25, 30, and 35% of the humeral length.

To evaluate the effect of humeral FOV on prediction accuracy, the network was retrained using images cropped to 35%, 30%, 25%, 20%, and 15% of the full humeral length, defined as a percentage of the distance from the humeral head center to the midpoint between the medial and lateral epicondyles (Fig. 4). Patients whose original proximal FOV did not encompass the required crop extent were excluded from the corresponding analyses, resulting in the removal of 14 patients for the 35% crop and 3 patients for the 30% crop. The networks were evaluated using the same performance metrics of mean absolute angular error and linear regression analysis. Prediction performance was compared between each cropped configuration and the original network. Pairwise Pearson correlations of signed prediction error were used to quantify patient-specific consistency across networks.

## Results

**Figure 5:**
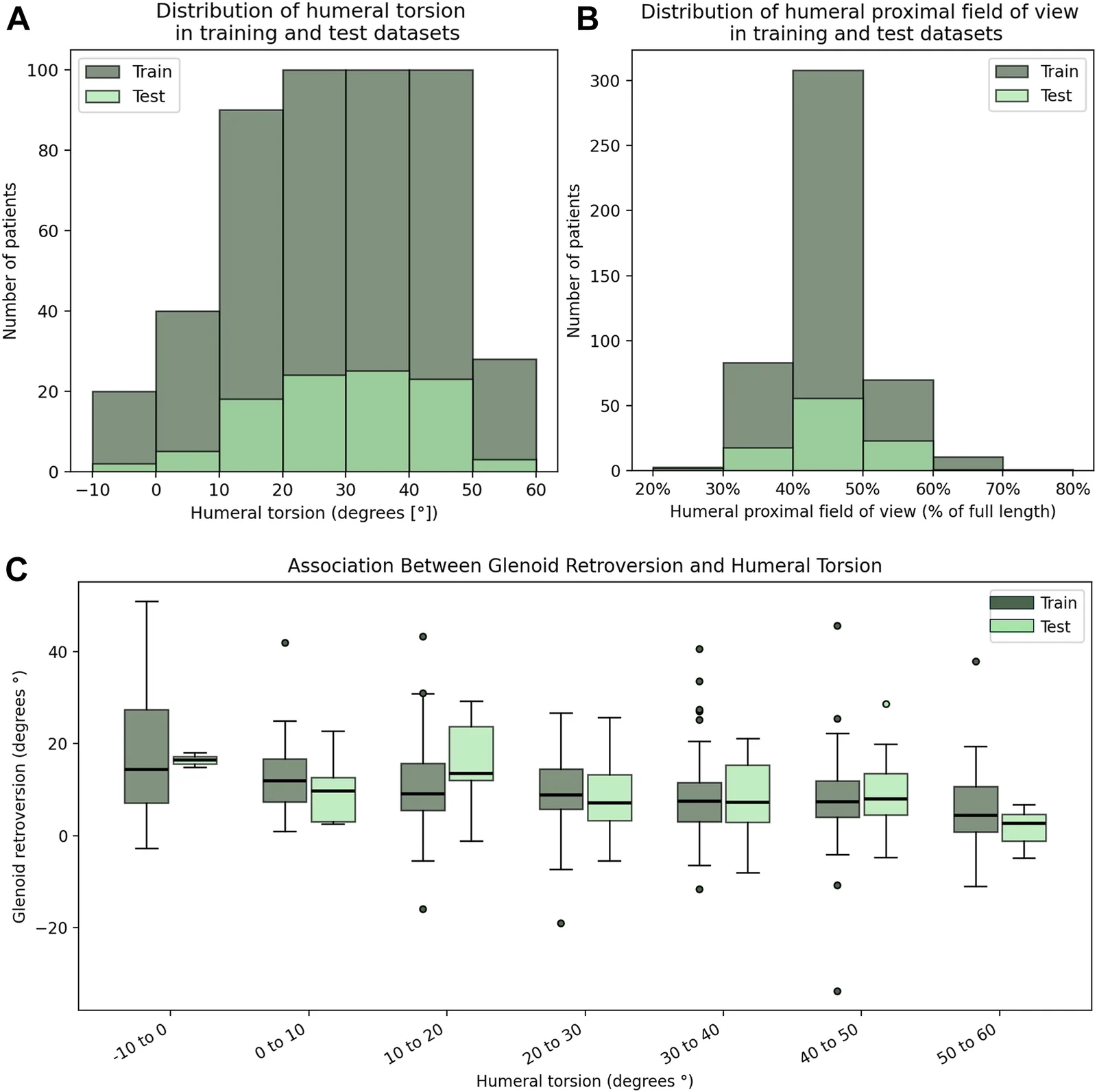
(**A**) Distribution of humeral torsion in the test and training data sets. (**B**) Distribution of proximal field of view expressed as a percentage of the full humeral length defined as the distance between the humeral head center and the midpoint between the medial and lateral humeral epicondyles. (**C**) Association between glenoid retroversion and humeral torsion per 10° humeral torsion bin.

The average humeral torsion of the test dataset was 29.28° ± 12.95° (range: −3.29° to 57.55°) compared to 28.06° ± 14.71° (range: −8.58° to 59.47°) of the training dataset (Fig. 5*A*). The mean FOV contained in the proximal humeral images was 45% ± 7% in the test dataset (range: 24%–61%) and 45% ± 7% in the training dataset (range: 25%–80%) (Fig. 5*B*). The glenoid retroversion decreased as posterior humeral torsion increased (Fig. 5*C*) in both the test and training data sets. There was no statistically significant difference between the glenoid version distributions between the test and training data.

The average humeral head sphericity of the test dataset was 50.93% ± 6.16% (range: 34.15% to 67.12%) and 50.72% ± 6.29% (range: 31.62% to 68.89%) for the training dataset.

**Figure 6:**
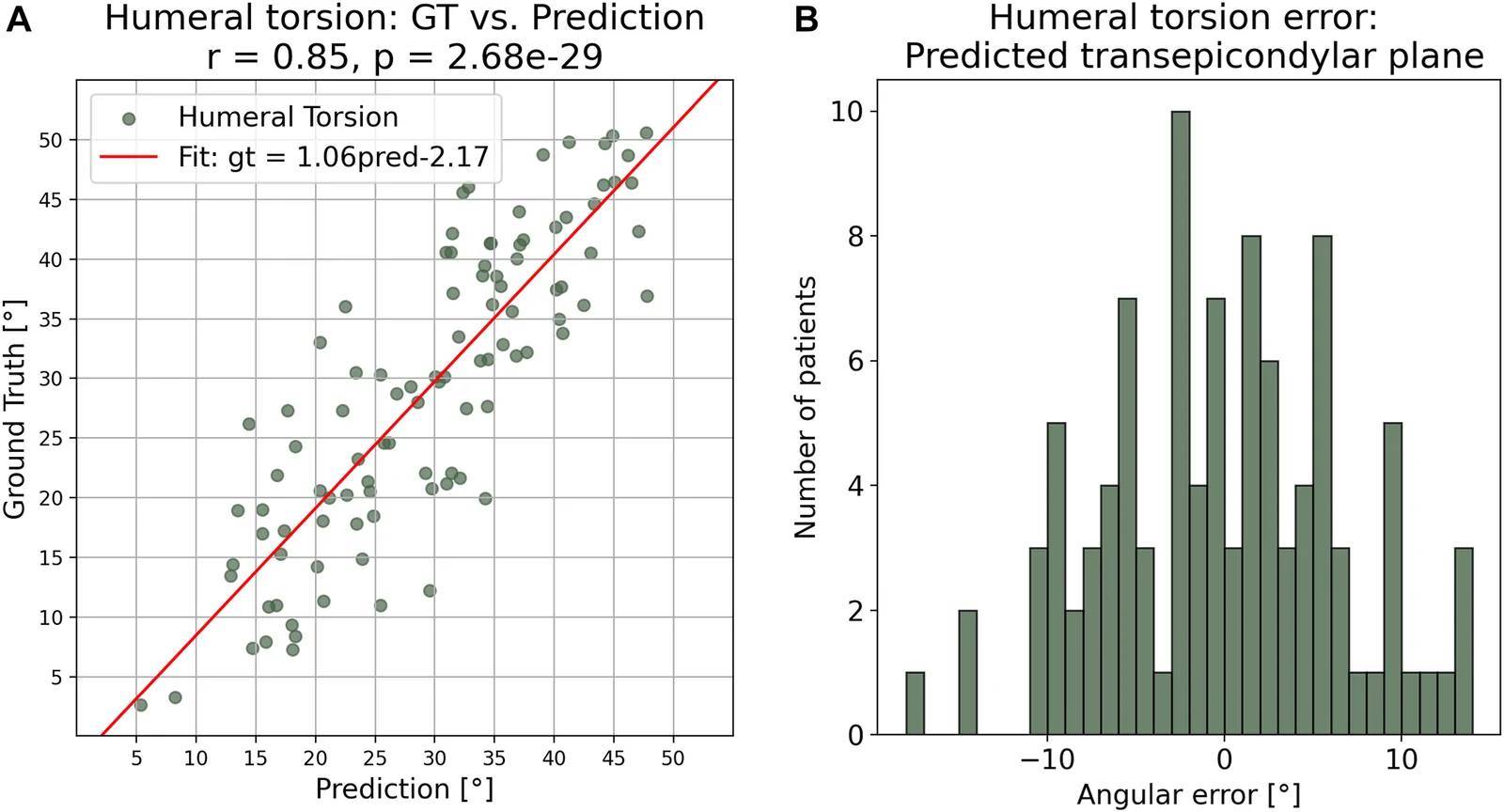
(**A**) Linear regression between humeral torsion calculated from the deep learning–predicted transepicondylar axis and the ground-truth (GT) values measured from elbow imaging. The correlation coefficient was r = 0.85. (**B**) Distribution of the signed angular prediction error between the GT and predicted measurements.

Using the orientations defined by the predicted planes, the mean angular error of the humeral normal axis prediction was 0.08° ± 0.2° (range: 0.0°–0.6°), and the mean absolute torsion prediction error was 5.35° ± 3.96° (range: 0.06°–17.37°) (Fig. 6*B* shows the relative error range). The linear regression analysis between predicted and ground truth humeral torsion revealed a strong linear association (R = 0.85, 95% confidence interval [CI]: [0.79–0.90]). The regression parameters of slope = 1.06 and intercept = −2.17 indicate an almost one-to-one correspondence between the predicted and ground-truth torsion values (Fig. 6*A*).

The error between the ground-truth torsion and the torsion calculated using the predicted transepicondylar axis showed no significant correlation with humeral head sphericity, the degree of torsion, or the FOV of the original CT scans.

Including osteophytes in the training network, the absolute torsion prediction error was 6.19 ± 3.95 (range: 0.09°-16.92°). Linear regression demonstrated a strong association between predicted and ground-truth torsion values (r = 0.82, 95% CI [0.74, 0.87]). Analysis of the pairwise Pearson correlations of the per-patient signed prediction error revealed a high correlation between networks trained with and without osteophytes (r = 0.85, 95% CI [0.79, 0.90]).

The network prediction performance for humeral torsion across different remaining proximal humeral FOV percentages is shown in Table II. Reducing the available humeral FOV was associated with a progressive increase in absolute torsion prediction error and a decrease in correlation with ground-truth measurements. Reducing the remaining FOV to 35% and 30% increased the mean absolute error to 6.23 ± 4.72° and 6.89 ± 5.09°, corresponding to increases of 0.88° ± 0.76° and 1.54° ± 1.13° in the standard deviation relative to the full FOV. At 25% of the humeral length, the mean absolute error increased by 1.66° ± 1.47° relative to the full proximal FOV (7.01 ± 5.43°), while the correlation decreased to R = 0.72 (95% CI [0.60, 0.80]); the per-patient signed prediction error remained moderately correlated with the full FOV network (R = 0.71, 95% CI [0.59, 0.79]). Further reductions below 25% resulted in higher errors (7.65 ± 5.77° at 20% and 8.26 ± 5.86° at 15%) and lower correlations with both ground truth (R = 0.62-0.67) and the full FOV network (R = 0.62–0.64).

**Table II:** Prediction performance of humeral torsion across different remaining proximal humeral field of view percentages.

Remaining humeral FOV [%]	Absolute torsion prediction error [°] (mean ± std, [min, max])	Correlation with ground truth and (95% CI)	Pair-wise signed error correlation (full vs. cropped FOV)
Full proximal field of view	5.35 ± 3.96 [0.06, 17.37]	R = 0.85, 95% CI [0.79, 0.90]	R = 1.00
35	6.23 ± 4.72 [0.12, 17.32]	R = 0.79, 95% CI [0.70, 0.85]	R = 0.86, 95% CI [0.79, 0.90]
30	6.89 ± 5.09 [0.12, 21.22]	R = 0.74, 95% CI [0.63, 0.82]	R = 0.76, 95% CI [0.66, 0.84]
25	7.01 ± 5.43 [0.19, 23.77]	R = 0.72, 95% CI [0.60, 0.80]	R = 0.71, 95% CI [0.59, 0.79]
20	7.65 ± 5.77 [0.12, 22.05]	R = 0.67, 95% CI [0.55, 0.77]	R = 0.62, 95% CI [0.47, 0.73]
15	8.26 ± 5.86 [0.03, 25.72]	R = 0.62, 95% CI [0.49, 0.73]	R = 0.64, 95% CI [0.50, 0.74]

*FOV*, field of view.

The table reports the mean and standard deviation (std) of the absolute torsion prediction error, the range of errors, the Pearson correlation coefficient with 95% confidence interval (95% CI) relative to the ground-truth measurements and the Pearson correlation coefficient with 95% confidence interval of the per-patient signed prediction error between cropped networks and the full proximal FOV network.

**Figure 7:**
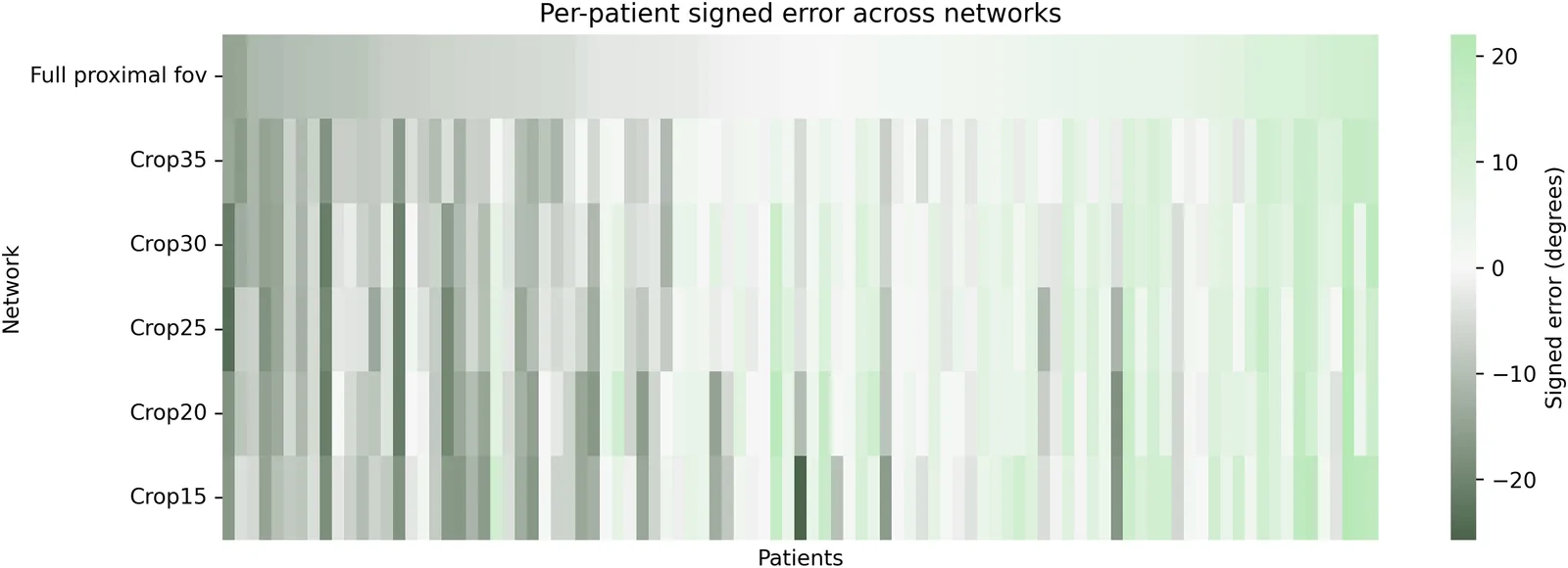
Heatmap of per-patient humeral torsion-signed prediction errors. The top row shows the baseline network with full available field of view (FOV). As the FOV is progressively cropped, the magnitude of the prediction errors changes; however, the sign of the errors is generally preserved across configurations.

Figure 7 shows a heatmap of per-patient signed humeral torsion prediction errors (relative to the ground truth) of the cropped network configurations and the full proximal FOV network. While smaller FOV networks show increased absolute prediction errors, the error sign remains consistent across models for each patient, indicating that the direction of the deviation from the ground truth is preserved. This consistency suggests that even networks trained on reduced fields of view retain stable patient-specific trends in humeral torsion estimation.

## Discussion

We present a deep learning–based method for predicting humeral torsion that demonstrates an almost one-to-one correspondence with ground-truth measurements, achieved through accurate estimation of the transepicondylar axis from 3D proximal humerus volumes. By representing the transepicondylar axis as a full 3D plane, the proposed approach reduces variability introduced by local surface irregularities and avoids reliance on discrete anatomical landmarks, which may not reside within a single, well-defined transverse plane.^9^ This formulation further enables the axis orientation to be encoded across the entire FOV, providing the network with comprehensive geometric context from which to infer torsional orientation based on the available anatomy.

When applied to predict the humeral normal axis, the mean angular orientation error relative to the ground-truth axis was less than 0.5°. This performance reflects the chosen representation, in which the image voxels were pre-aligned with the target plane and the relevant anatomical context is contained within the FOV. When applied to predict the orientation of the plane representing the epicondylar axis, information not explicitly contained within the FOV, our resulting network plane prediction resulted in a humeral torsion prediction error of 5.35° ± 3.96°, lower than the previously reported mean bilateral difference in torsion of 6.9° ± 5.3°.^28^ The method also demonstrated a substantially stronger correlation with ground truth humeral torsion (R = 0.85) compared to prior predictions based on proximal humeral anatomy using the bicipital groove (R = 0.33–0.42)^5^^,^^18^ and the intertubercular sulcus (R = -0.37)^8^ indicating improved predictive performance.

This method demonstrated robustness to humeral osteophytes, with the predicted transepicondylar plane yielding a mean absolute torsion error within 1° of the baseline network. The high pairwise correlation of signed errors between networks trained with and without osteophytes (R = 0.85) indicates strong per-patient consistency, suggesting that error patterns are largely preserved and minimally affected by the inclusion of osteophytes.

Analysis of the effect of proximal FOV on deep learning–based humeral torsion prediction showed a gradual degradation in performance as the available humeral anatomy was reduced. Progressive cropping was associated with increased absolute prediction error and a concomitant decrease in correlation with ground-truth torsion, indicating that additional proximal humeral context contributes to more accurate and stable axis orientation estimation. Nevertheless, despite higher inaccuracies, humeral torsion prediction was still possible even with a small FOV with the preservation of per-patient signed error patterns across all FOV configurations. This suggests that the networks relied on consistent anatomical cues, even when these were only partially available. Notably, although predictions deteriorated substantially when only 10% of the FOV remained, the network only failed with 5% remaining humerus, in which case barely any bicipital groove or medial-lateral anatomical variation was included. This finding provides computational support for long-standing anatomical and biomechanical hypotheses, suggesting that native humeral torsion arises from shear forces generated by the rotator cuff musculature, which inserts proximal to the humeral epiphysis. These forces act during growth and during osseous adaptation to mechanical stress to alter the rotational relationship between the proximal epiphysis and the diaphysis, ultimately shaping the adult humeral torsional alignment.^7^^,^^17^^,^^25^^,^^26^ Studies have, however, also shown that increased mechanical loading and biomechanical demands, such as those encountered in throwing sports, can modulate humeral torsion, resulting in higher torsion angles in throwing athletes than nonathletes.^7^^,^^26^^,^^29^ These findings suggest a dynamic and potentially degenerative component to the development of humeral torsion. In the context of osteoarthritis, Walch B glenoids have been shown to have reduced humeral retrotorsion compared to healthy glenoids.^22^ Such variability may contribute to the observed prediction uncertainty of the network. However, further investigation is needed to clarify the underlying mechanisms and their relationship to osteoarthritis disease progression.

The precise clinical threshold for acceptable humeral torsion prediction error in rTSA planning has not yet been established by prospective outcome data. Nevertheless, indirect evidence from biomechanical and clinical studies allows the clinical significance of our results to be contextualized. For improved outcomes of rTSA including balanced internal and external rotation, biomechanical and clinical studies support matching implant retrotorsion to native humeral torsion,^19^^,^^20^ highlighting the importance of accurate retroversion measurement during implant planning. Because humeral torsion directly influences the determination of humeral component version,^15^^,^^21^^,^^27^ failure to account for it can result in large discrepancies between planned and executed implant orientation, reported in some cases up to 40°.^21^ These findings suggest that the torsion estimation accuracy of 5.35° ± 3.96° achieved by the trained network is clinically relevant for 3D implant planning. Compared to previously described methods predicting humeral torsion when distal landmarks are unavailable, our method demonstrated significant improvement.^5^^,^^8^^,^^18^

A limitation of this study is that highly dysplastic humeri were excluded from network training. To our knowledge, no universally accepted clinical classification of humeral head dysmorphology currently exists. Thus, exclusion was performed by visual inspection rather than by a formal grading system. In cases of severe dysplasia or large osteophytes, the humeral head normal axis, defined as the humeral normal axis perpendicular to the articular surface of a best-fit sphere, becomes geometrically ill-defined: When the articular surface deviates substantially from a sphere, this axis no longer carries a consistent anatomical meaning, and the torsion angle derived from the relationship between it and the epicondylar plane loses clinical interpretability regardless of measurement accuracy. For this reason, 212 patients were excluded from this study. Nonetheless, training of the network was performed using as broad a spectrum of torsion values (−8.58° to 59.47°), and the network was tested for humeral torsion prediction across a similarly wide range (−3° to 58°), including cases with mild to moderate humeral head dysplasia and osteophyte burden. This ensured that the network was trained and validated on a morphologically diverse population representative of the range encountered in routine shoulder arthroplasty practice. The multi-vendor, multi-protocol nature of the dataset, spanning five CT scanner vendors and resolutions from 0.20 to 2.00 mm, provides a degree of implicit cross-scanner robustness, but prospective validation in an independent cohort will be necessary to confirm generalizability before clinical deployment. Additionally, future work could further explore the exclusion of highly dysplastic humeri discussed above. In such cases, estimating torsion directly from the morbid anatomy may be challenging or of limited clinical relevance, as severe deformity can obscure the anatomical reference axis. One possible approach may be to first predict or reconstruct the premorbid humeral head geometry, providing a more consistent reference surface for axis determination. Training on larger datasets that include highly dysplastic cases, potentially in combination with such a reconstruction step, may help enable torsion estimation in substantially altered anatomy. Finally, beyond the shoulder, this general framework approach could be extended to other long bones such as the femur, which similarly has a limited FOV of the knee in standard clinical imaging, as well as testing the algorithm on other imaging modalities.

## Conclusion

We present a deep learning–based approach for predicting humeral torsion directly from 3D proximal humerus volumes, without reliance on distal anatomy. By representing the transepicondylar axis as a full 3D plane extending throughout the available FOV, the orientation of the network-predicted plane enables torsion estimation with near one-to-one correspondence to ground-truth measurements while reducing sensitivity to local surface irregularities. The method demonstrates clinically relevant performance, exhibiting a strong linear correlation with ground-truth values (r = 0.85) and a mean prediction error of 5.35° across a torsion range of −3° to 58°, outperforming prior approaches based on proximal landmark definitions. These findings suggest that torsional information is largely encoded in proximal humeral morphology, supporting existing anatomical and biomechanical hypotheses regarding the development of humeral torsion.

## Disclaimers:

Funding: Open access funding was provided by the University of Bern. This research was funded by the Innosuisse project number 116.166 IP-LS.

Conflicts of interest: The authors, their immediate families, and any research foundations with which they are affiliated have not received any financial payments or other benefits from any commercial entity related to the subject of this article.
